# Challenges experienced by early career researchers in Africa

**DOI:** 10.2144/fsoa-2020-0012

**Published:** 2020-03-31

**Authors:** Tijjani Salihu Shinkafi

**Affiliations:** 1 Department of Biochemistry, Faculty of Science, Usmanu Danfodiyo University Sokoto, PMB 2346, Sokoto, Nigeria

**Keywords:** African scientists, career progression, early career researchers, funding, mentorship

## Who are early career researchers?

Early career researchers (ECRs) can be on a tenure track position or not. Tenured track ECRs can be in the research institutes, universities, polytechnics or colleges. In African universities, the rank of ECRs ranges from assistant lecturers to lecturer or occasionally senior lecturers (in very rare circumstances). ECRs are called postdocs or postdoctoral fellows in developed countries. They are generally inexperienced researchers who require a lot of guidance, support, mentorship and motivation to ascend through difficult and challenging job, requiring the ability to maintain a balance between teaching, research and administrative responsibilities [1]. African institutions do not pay much attention to ECRs; as a result, they easily get frustrated and lose interest in the job. Here I will discuss some of the challenges faced by the ECRs in Africa, with a focus on Nigeria.

### Support, mentorship & guidance

Most ECRs in Africa do not get the required training, mentorship and guidance from experienced scholars in their institution upon assumption of duty. In a study conducted in Malawi with respondents across many African countries, it was found that lack of funding and mentorship from senior scholars are the major challenges encountered by the ECRs of the continent [2]. This orientation is essential especially at the assumption of duty level. At this time ECRs need guidance to be able to overcome some of the challenges they may encounter. An orientation program is essential to young academics to enable them to write good papers and seek their publication in relevant peer-reviewed journals. In Europe, senior academics usually appear to be more visible in providing guidance and mentorship to ECRs [3,4].

### Funding

Research funding in Africa is very limited and the emphasis is given mostly to senior scholars, that is, the associate professors and professors. Taking Nigeria as an example, funding agencies do not give priority attention to ECRs. In most countries, institutions and funding agencies accord priority attention to ECRs to enable them to settle down and establish themselves as researchers. Institution-based research funds provided by the Tertiary Institution Trust Fund (Tetfund) were reactivated recently and allow Nigerian university researchers to avail of a maximum of 2 million naira for research. However, when you convert this amount to dollars it is around US$5000, an insufficient amount with which to conduct science-based research in a laboratory that is not well established. Similarly, the National Research Fund, also provided by the Tetfund, allows a Nigerian to attract a grant of up to 50 million naira at a time. However, this was not designed to accommodate ECRs. One needs to be a senior lecturer and above to be eligible to get the National Research Fund. Researchers, especially in the sciences, engineering and medical sciences, cannot publish a ‘good’ paper in a reputable journal without grants or funding [5]. Researchers, who cannot publish, perish.

### Laboratory space, chemicals & reagents

Ideally, a university lecturer with a PhD degree is supposed to have a well-equipped state-of-the-art laboratory where his students can work. In the laboratory, you can find one or two or more postdocs and some PhD students conducting research. The postdocs assist in overseeing the PhD students and the laboratory on behalf of the supervisor who serves as their overall mentor. There is no way that laboratory can be furnished with state-of-the-art facilities without funds. The professor, in collaboration with their students (such as the ECRs) and their colleagues, must be able to write good proposals for grants. By this way, all the needed facilities such as equipment, chemicals and reagents as well as consumables need to be purchased for immediate use in the laboratory. This scenario is completely absent in Nigeria. Similarly, in many African countries, the acquisition of reagents or chemicals is a problem. Most companies do not have representatives in most African countries, making supply of important chemicals to Africa difficult.

### Adjustment after PhD

Most Nigerian PhD holders in the universities, polytechnics, colleges or research institutes in Nigeria were sponsored either through Tetfund, the Petroleum Technology Development Fund or other funding from abroad. The kind of research facilities and techniques they might have used during their PhD elsewhere are often unavailable in Nigeria. The major challenge they therefore encounter first is how to adjust and how to conduct research in this kind of environment where even the light might not be stable.

### Difficulty in acquiring foreign postdoctoral or visiting fellowships

Most ECRs who did their PhD at African universities finds it difficult to get a postdoctoral fellowship outside their country. The reason for this is their inability to find a suitable host mentor or supervisor who can be convinced to work with them. Writing a good statement of purpose, proposal and curriculum vitae is a key to success in getting those postdoctoral fellowships as well as harnessing the skills acquired during the PhD.

### The burden of administrative responsibilities

Academic staff in most cases in African universities are burdened by too many administrative responsibilities. For example, in my department presently I am both the project coordinator (for undergraduate research projects), seminar coordinator (departmental seminars for both students and academic staff) and students’ industrial training experience scheme coordinator, in addition to being the new teaching laboratories coordinator for the Faculty of Science and deputy coordinator for the students’ industrial training experience scheme unit of the university. I am also a member/secretary of numerous university committees besides the primary role of teaching. This leaves little time to conduct research, write good papers and apply for grants.

## Consequences of a lack of support for ECRs

The consequences of the lack of attention to these researchers are enormous. There is a possibility of them leaving the university at an early stage for a lucrative job. Recently, I have seen several of my colleagues with PhDs leave universities to go into banking, insurance or other positions that pay very well. However, every PhD holder would prefer to remain in the university environment provided the condition and atmosphere is conducive for them, inclusive of a good salary package. In the university environment, PhD holders could have a lot of future prospects. Unfortunately, the current system does not support them to grow and become authorities in their domain. As a result, due to this lack of support, in the future the universities and research institutes of Nigeria will lack renowned experts who are expected to lead the continent in research. Consequently, the quality of research conducted in these kinds of institutes will diminish over time because of the lack of experts.

## Recommendations

The academic environment must be tailored toward generating experts in several areas of knowledge. This is only possible when ECRs are given the required support, mentorship and guidance to become established in their fields of interest. Senior academics must volunteer to provide the prerequisite training and guidance to young upcoming researchers who will likely take over from them in future.

Funding agencies should consider supporting ECRs who have been unable to get tenure track positions in efforts to take a postdoctoral fellowship opportunity. In Nigeria, Petroleum Technology Development Fund and TetFund and other funding agencies can support young PhD holders who are not employed in enrollment for postdoctoral training upon completion of PhD. This would certainly reduce the unemployment rate in the country. Few universities in Africa offer postdoctoral opportunities, yet this is very common in universities in developed countries. Similar opportunities have been advertised in South Africa. The African Union has also started as of last year the African Postdoctoral Training Initiative, through the African Academy of Science, where in collaboration with the NIH and the Bill and Melinda Gates Foundation they sponsor at least ten ECRs of African origin to undertake a 2-year postdoctoral training with the NIH, with a view to train them to become experts in their area of interest. This measure taken by the African Union is expected to build bridges, thereby creating room for lasting connections between partner organizations (that is, the NIH and Bill and Melinda Gates Foundation) with African scientists (who are likely to take a lead in the research issues of the continent) and their institutions.

## Conclusion

Whether in Africa or in developed countries, ECRs experience challenges during career progression. Provision should be there to support these scholars to become fully established after completing their PhD otherwise the purpose of obtaining the training will not be achieved. The major difficulties that are peculiar to ECRs in Africa are the lack of mentorship, funding and laboratory space, in addition to the challenges faced elsewhere such as lack of enough time for research, publishing and career progression. It is therefore necessary for the institutions in Africa to make provision for laboratory space as well as reduce the workload for ECRs to enable them to be productive and meet their potential. Similarly, the funding agencies must consider ECRs when planning funding to enable the conduct of good research.
